# Association of high fibrinogen to albumin ratio with long-term mortality in patients with spontaneous intracerebral hemorrhage

**DOI:** 10.3389/fneur.2024.1412804

**Published:** 2024-07-19

**Authors:** Shiping Chen, Yu Zhang, Yangchun Xiao, Xin Cheng, Liyuan Peng, Yixin Tian, Tiangui Li, Jialing He, Pengfei Hao, Weelic Chong, Yang Hai, Chao You, Fang Fang

**Affiliations:** ^1^Department of Neurosurgery, Clinical Medical College and Affiliated Hospital of Chengdu University, Chengdu, Sichuan, China; ^2^Department of Neurosurgery, West China Hospital, Sichuan University, Chengdu, Sichuan, China; ^3^Department of Neurosurgery, The First People's Hospital of Longquanyi District Chengdu, Chengdu, Sichuan, China; ^4^Department of Neurosurgery, The Second Affiliated Hospital of Guangzhou Medical University, Guangzhou, Guangdong, China; ^5^Department of Neurosurgery, Shanxi Provincial People’s Hospital, Taiyuan, Shanxi, China; ^6^Department of Medical Oncology, Thomas Jefferson University, Philadelphia, PA, United States; ^7^Sidney Kimmel Medical College, Thomas Jefferson University, Philadelphia, PA, United States; ^8^Department of Neurosurgery, Sichuan Friendship Hospital, Chengdu, Sichuan, China

**Keywords:** intracerebral hemorrhage, fibrinogen, albumin, ratio, long-term mortality

## Abstract

**Background:**

The association between fibrinogen-to-albumin ratio (FAR) and in-hospital mortality in patients with spontaneous intracerebral hemorrhage (ICH) has been established. However, the association with long-term mortality in spontaneous ICH remains unclear. This study aims to investigate the association between FAR and long-term mortality in these patients.

**Methods:**

Our retrospective study involved 3,538 patients who were diagnosed with ICH at West China Hospital, Sichuan University. All serum fibrinogen and serum albumin samples were collected within 24 h of admission and participants were divided into two groups according to the FAR. We conducted a Cox proportional hazard analysis to evaluate the association between FAR and long-term mortality.

**Results:**

Out of a total of 3,538 patients, 364 individuals (10.3%) experienced in-hospital mortality, and 750 patients (21.2%) succumbed within one year. The adjusted hazard ratios (HR) showed significant associations with in-hospital mortality (HR 1.61, 95% CI 1.31–1.99), 1-year mortality (HR 1.45, 95% CI 1.25–1.67), and long-term mortality (HR 1.45, 95% CI 1.28–1.64). Notably, the HR for long-term mortality remained statistically significant at 1.47 (95% CI, 1.15–1.88) even after excluding patients with 1-year mortality.

**Conclusion:**

A high admission FAR was significantly correlated with an elevated HR for long-term mortality in patients with ICH. The combined assessment of the ICH score and FAR at admission showed higher predictive accuracy for long-term mortality than using the ICH score in isolation.

## Introduction

Spontaneous Intracerebral Hemorrhage (ICH) is a severe condition associated with high mortality and disability rates, thus imposing long-term escalation burdens on both individuals and society (1–3). To compensate for the lack of effective treatments for ICH, various prognostic models have been developed for predicting short-term mortality and functional outcomes after ICH (4, 5). However, accurately predicting long-term outcomes still poses a challenge. Thus, it becomes imperative to identify additional reliable predictors of ICH outcomes.

A significant body of clinical and experimental evidence suggests that inflammation is a crucial factor in predicting outcomes of ICH (6, 7). Studies have shown that fibrinogen plays a pivotal role in inflammation (8, 9). Elevated levels of fibrinogen have been linked to the development and enlargement of ICH (10, 11), and exacerbated neuronal damage (12). Moreover, fibrinogen has been recognized as a promising marker for assessing the severity and prognosis of ICH (13) On the other hand, albumin serves as a vital parameter for evaluating patients’ nutritional status and systemic inflammation (14, 15). Lower levels of serum albumin have been linked to larger hematoma volumes, heightened complication risks, and increased mortality rates in individuals diagnosed with ICH (16, 17).

The fibrinogen to albumin ratio (FAR) is a new inflammatory biomarker that has shown promising results in predicting prognosis for various diseases (18–21). Evidence has indicated an association between admission FAR and in-hospital mortality rates in individuals with ICH (11). However, the association between the FAR and long-term mortality remained unclear. Therefore, the aim of our cohort study is to examine the association between FAR and long-term mortality in patients with ICH.

## Methods

### Study design

A total of 3,538 consecutive patients diagnosed with ICH were retrospectively identified at West China Hospital, Sichuan University, from December 2010 to August 2019 for this study. The data utilized in this study was obtained from the hospital’s electronic medical records. To gather data on patient survival, we utilized the Household Registration Administration System of the People’s Republic of China. This system keeps track of current mortality records for residents, providing us with accurate and up-to-date information. The study (No. 2022-705) received approval from the institutional review boards of the ethics committees at West China Hospital. Informed consent was waived considering the nature of the clinical audit. The study followed the STROBE criteria and adhered to the ethical principles laid out in the Declaration of Helsinki 1964.

### Patient selection

All patients included in our study were diagnosed with spontaneous ICH. The diagnosis was confirmed through brain magnetic resonance imaging or computed tomography at admission, and further assessed by a neurologist during hospitalization. Our study specifically focused on patients more than 18 years old.

Patients were excluded from the study based on specific criteria: (1) patients with ischemic stroke accompanied by hemorrhagic transformation, traumatic brain injury, subarachnoid hemorrhage from cerebral aneurysm, bleeding from ruptured vascular malformation, and hemorrhage due to coagulation abnormalities in the brain, or other conditions that were distinct from primary ICH; (2) serum fibrinogen or albumin data were not available within 24 h of admission; (3) invalid personal identification numbers or inaccurate survey data from the Household Registration Administration System.

### Clinical characteristics and laboratory data collection

We collected demographic characteristics such as age, gender, current smoking status, and alcohol consumption. Medical history details, including hypertension, diabetes, oral anticoagulant or antiplatelet drugs, chronic kidney disease, and chronic liver disease were also recorded. Additionally, we gathered data on systolic blood pressure and diastolic blood pressure at admission, hematoma location (infratentorial and supratentorial), maximum hematoma volume during hospitalization [calculated using the formula ABC/2 (22)], and the presence of an intraventricular hematoma. The severity of ICH at admission was assessed using the Glasgow Coma Scale (GCS) score, dividing it into a score < 9 group, a score 9–12 group, and a score ≥ 13 group. The craniotomy involved a hematoma evacuation. Furthermore, we collected general laboratory tests, including neutrophil count, activated partial thromboplastin time, glucose levels, and platelet count the first time within 24 h of admission.

### Exposure

In our study, all serum fibrinogen and serum albumin samples were collected within 24 h of admission and participants were divided into two groups according to the median of FAR, specifically into the low FAR group (FAR ≤0.07) and the high FAR group (FAR >0.07). Furthermore, we also collected the latest serum fibrinogen and serum albumin samples before the discharge of surviving patients. Based on the receiver operating characteristic (ROC) analysis for long-term mortality, we classified the discharged survivors into two groups using the FAR discriminative cut-off value.

### Outcomes

The primary outcome of this study was long-term mortality, specifically defined as all-cause mortality at the longest follow-up period. The second outcome was in-hospital mortality, defined as all-cause mortality before discharge, and 1-year mortality, defined as all-cause mortality within 1 year after discharge. The data regarding deaths during the follow-up were sourced from the Household Registration Administration System of China, recognized for its precise death records and reliable assessment (23).

### Statistical analysis

The study presented continuous data, comprising age, GCS score, hematoma volume, neutrophil count, activated partial thromboplastin time, glucose levels, and platelet count, in terms of means and standard deviations. Statistical analysis involved comparing continuous variables using the student’s *t*-test and Mann–Whitney *U*-test. Categorical data were examined by computing frequencies and percentages, and then comparing them using either the chi-square test or Fisher’s exact test. To address any missing data, multiple imputation techniques were implemented.

The Cox proportional hazards model was used for analyzing independent prognostic factors for long-term mortality in both univariate and multivariate analyses. The Cox proportional hazards model included baseline variables, relevant demographics, and laboratory tests selected based on prior research and clinical knowledge (4). A two-sided *p* value <0.05 was set as the threshold for statistical significance.

The hazard ratios (HR) of long-term mortality risk associated with FAR in ICH patients were evaluated using restricted cubic spline analysis. The Kaplan–Meier method was utilized to estimate survival curves, and the log-rank test was applied to compare them. Additionally, receiver operating characteristic (ROC) curves were developed to evaluate the area under the curve (AUC) based on ICH score, reflecting the test’s sensitivity and specificity. The ICH score defined as a clinical grading scale including GCS, age, ICH volume, IVH, and the hemorrhage location (infratentorial or supratentorial). Additional subgroup analyses were performed according to age (≤65 years and > 65 years), gender, smoking habits, alcohol consumption, hypertension, diabetes, chronic kidney disease, GCS scores (<9 and ≥ 9), systolic blood pressure, location of hematoma (infratentorial and supratentorial), hematoma volume (≤30 mL and > 30 mL), and presence of intraventricular hematoma to evaluate outcome heterogeneity. R software (version 4.3.2; R Foundation for Statistical Computing) was utilized for all statistical analyses.

## Results

**Figure 1:**
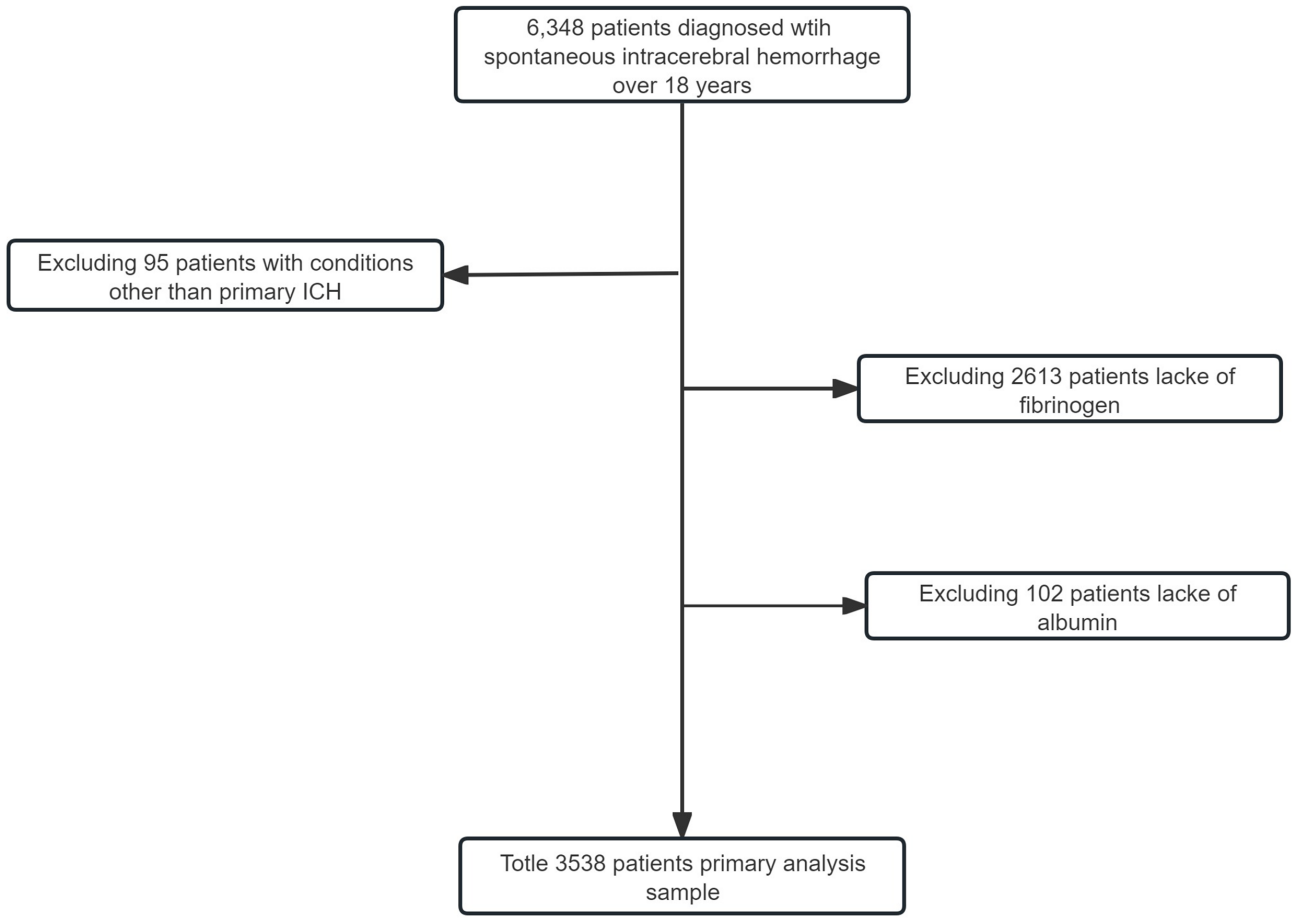
Flow chart of enrollment.

A total of 6,348 patients aged over 18 years with a diagnosis of ICH at West China Hospital were included in the study (Figure 1). Exclusions involved 95 patients with conditions other than primary ICH, 2,613 patients lacking fibrinogen, 102 patients lacking albumin upon admission, and one patient with unacceptable survey information from the Household Registration Administration System. Consequently, our analysis focused on 3,538 ICH patients, with 364 patients (10.3%) experiencing in-hospital mortality, 750 patients (21.2%) succumbing within one year, and 1,013 patients (28.6%) experiencing long-term mortality. Table 1 outlines the baseline characteristics of the groups. Patients with a higher FAR were observed to be older, more likely to smoke, have diabetes, and suffer from chronic kidney disease. Furthermore, these patients tended to present with larger hematoma volumes, lower GCS scores, increased platelet counts, elevated admission blood glucose levels, increased fibrinogen levels, and decreased albumin levels.

**Table 1:** Baseline characteristics of the patients.

Characteristics	Fibrinogen to albumin ratio			*p*	Miss value (*n*, %)
	Overall (*n* = 3,538)	Low FAR* (FAR≤0.07, *n* = 1769)	High FAR* (FAR >0.07, *n* = 1769)		
Demographics					
Age, years, mean (SD)	56.35 (14.59)	55.31 (14.69)	57.39 (14.43)	<0.001	
Female, *n* (%)	1,140 (32.2)	593 (33.5)	547 (30.9)	0.105	
Smoking, *n* (%)	906 (25.6)	421 (23.8)	485 (27.4)	0.015	
Alcohol, *n* (%)	1,110 (31.4)	548 (31.0)	562 (31.8)	0.638	
Medical history, *n* (%)					
Hypertension	2,554 (72.2)	1,258 (71.1)	1,296 (73.3)	0.165	
Diabetes	412 (11.6)	163 (9.2)	249 (14.1)	<0.001	
Chronic kidney disease	109 (3.1)	31 (1.8)	78 (4.4)	<0.001	
Chronic liver disease	0.04 (0.20)	0.03 (0.17)	0.05 (0.22)	0.008	
SBP, mmHg, mean (SD)	159 (32.7)	156 (32.4)	158 (33.0)	0.061	37 (1.0)
DBP, mmHg, mean (SD)	99 (223.3)	104 (314.8)	93 (20.1)	0.122	37 (1.0)
Hematoma characteristics					
Infratentorial hematoma, *n* (%)	748 (21.1)	359 (20.3)	389 (22.0)	0.232	
Size of hematoma, cm^3^, mean (SD)	23.06 (27.69)	24.44 (29.63)	21.57 (25.35)	0.004	468 (13.2)
Intraventricular hematoma, *n* (%)	789 (22.3)	385 (21.8)	404 (22.8)	0.467	
GCS score, *n* (%)				0.001	
3–8 score	1,321 (37.3)	607 (34.3)	714 (40.4)		
9–12 score	879 (24.8)	459 (25.9)	420 (23.7)		
13–15 score	1,338 (37.8)	703 (39.7)	635 (35.9)		
Craniotomy, *n* (%)	1,005 (28.4)	579 (32.7)	426 (24.1)	<0.001	
Laboratory tests, mean (SD)					
Platelet, 10^9^/L	158.6 (74.1)	155.1 (71.6)	162.1 (76.4)	0.005	28 (0.8)
Lymphocyte count, 10^9^/L	1.11 (0.70)	1.12 (0.77)	1.09 (0.61)	0.117	31 (0.9)
Activated partial thromboplastin time, s	28.79 (9.38)	28.49 (10.83)	29.08 (7.66)	0.061	
Blood glucose, mmol/L	7.54 (3.20)	7.37 (2.91)	7.71 (3.47)	0.002	
Neutrophil count, 10^9^/L	9.07 (5.31)	9.05 (6.11)	9.09 (4.38)	0.797	31 (0.9)
Fibrinogen, g/L	3.02 (1.25)	2.18 (0.53)	3.86 (1.20)	<0.001	
Albumin, g/L	38.56 (6.35)	40.60 (5.81)	36.52 (6.20)	<0.001	
Admission Fibrinogen to Albumin Ratio, mean (SD)	0.08 (0.04)	0.05 (0.01)	0.11 (0.04)	<0.001	

SBP, systolic blood pressure; DBP, Diastolic Blood Pressure; GCS, Glasgow Coma Scale. The student’s *t*-test was used to compare age, SBP, size of hematoma, platelet, lymphocyte count, activated partial thromboplastin time, blood glucose, neutrophil count, albumin, admission fibrinogen to albumin ratio. The chi-square test was used to compare sex, smoking, alcohol, hypertension, diabetes, chronic kidney disease, chronic liver disease, infratentorial hematoma, intraventricular hematoma, intraventricular hematoma. *Patients were divided into two groups according to the median of FAR (0.07).

The primary outcomes of the study were analyzed using multivariate Cox regression, as presented in Table 2. The HR adjusted for potential confounders revealed significant associations with in-hospital mortality (HR 1.61, 95% CI 1.31–1.99), 1-year mortality (HR 1.45, 95% CI 1.25–1.67), and long-term mortality (HR 1.45, 95% CI 1.28–1.64). Furthermore, the HR for long-term mortality remained statistically significant at 1.47 (95% CI, 1.15–1.88) even after excluding patients with 1-year mortality. The factors included in the multivariable logistic regression analysis were age, Glasgow Coma Scale (GCS) score, hematoma location, hematoma volume, and presence of intraventricular hematoma (Supplementary Table S1). The adjusted HR for long-term mortality remained significant even after excluding patients with 1-year mortality (Supplementary Table S2). The association between discharge fibrinogen to albumin ratio and long-term mortality were shown in Table 3. The discharged survivor patients were categorized into two groups: the low FAR group (FAR ≤0.09) and the high FAR group (FAR >0.09) using the FAR discriminative cut-off value of 0.09 based on the ROC analysis for long-term mortality. The HR adjusted for potential confounders revealed significant associations with long-term mortality (HR 1.28, 95% CI 1.08–1.50), and excluding patients with 1-year mortality (HR 1.66, 95% CI 1.28–2.16). Albumin and fibrinogen levels were both significantly associated with long-term mortality. Albumin had HRs of 0.61 (95% CI 0.54–0.70) and 0.52 (95% CI 0.41–0.68) when excluding patients with 1-year mortality (Supplementary Table S3). Fibrinogen showed HRs of 1.22 (95% CI 1.07–1.38) and 1.45 (95% CI 1.12–1.86) when excluding patients with 1-year mortality (Supplementary Table S4).

**Table 2:** The association between fibrinogen to albumin ratio and mortality using multivariate Cox regression.

Outcomes	Events/Total, *n* (%)	Cox regression unadjusted HR	*p*	Cox regression adjusted HR	*p*
Mortality before discharge					
Continues	364/3538 (10.3%)	1.09 (1.08–1.11)	<0.001	1.07 (1.05–1.09)	<0.001
Low FAR	145/1769 (8.2%)	1 [Reference]		1 [Reference]	
High FAR	219/1769 (12.4%)	1.61 (1.31–1.99)	<0.001	1.30 (1.05–1.60)	0.017
1-year mortality					
Continues	750/2222 (33.8%)	1.08 (1.06–1.09)	<0.001	1.05 (1.03–1.06)	<0.001
Low FAR	322/1123 (28.7%)	1 [Reference]		1 [Reference]	
High FAR	428/1099 (38.9%)	1.45 (1.25–1.67)	<0.001	1.18 (1.02–1.37)	0.023
Long-term Mortality in all patients					
Continues	1013/2222 (45.6%)	1.08 (1.06–1.09)	<0.001	1.05 (1.04–1.07)	<0.001
Low FAR	435/1123 (38.7%)	1 [Reference]		1 [Reference]	
High FAR	578/1099 (52.6%)	1.45 (1.28–1.64)	<0.001	1.21 (1.07–1.38)	0.003
Long-term mortality in 1-year survivors					
Continues	263/1472 (17.9%)	1.08 (1.05–1.11)	<0.001	1.06 (1.03–1.09)	<0.001
Low FAR	113/801 (14.1%)	1 [Reference]		1 [Reference]	
High FAR	150/671 (22.4%)	1.47 (1.15–1.88)	0.002	1.31 (1.02–1.68)	0.034

The model was adjusted for age, Glasgow Coma Scale score, hematoma location, hematoma volume, intraventricular hematoma, chronic liver disease, and craniotomy. FAR, Fibrinogen to Albumin Ratio.

**Table 3:** The association between discharge fibrinogen to albumin ratio and mortality using multivariate Cox Regression.

Outcomes	Events/Total, *n* (%)	Cox regression Unadjusted HR	*p*	Cox regression Adjusted HR*	*p*
1-year mortality					
Continues	382/1816 (21.0%)	1.05 (1.04–1.07)	<0.001	1.03 (1.01–1.05)	0.01
Low FAR (FAR ≤0.09)	198/1098 (18%)	1 [Reference]		1 [Reference]	
High FAR (FAR >0.09)	184/718 (25.6%)	1.47 (1.20–1.80)	<0.001	1.07 (0.86–1.32)	0.546
Long-term mortality in all patients					
Continues	636/1816 (35.0%)	1.06 (1.05–1.07)	<0.001	1.04 (1.02–1.05)	<0.001
Low FAR (FAR ≤0.09)	316/1098 (28.8%)	1 [Reference]		1 [Reference]	
High FAR (FAR >0.09)	320/718 (44.6%)	1.69 (1.44–1.97)	<0.001	1.28 (1.08–1.50)	0.004
Long-term mortality in 1-year survivors					
Continues	254/1434 (17.7%)	1.08 (1.06–1.11)	<0.001	1.06 (1.03–1.09)	<0.001
Low FAR (FAR ≤0.09)	118/900 (13.1%)	1 [Reference]		1 [Reference]	
High FAR (FAR >0.09)	136/534 (25.5%)	2.07 (1.62–2.65)	<0.001	1.66 (1.28–2.16)	<0.001

Based on the ROC analysis for long-term mortality, the discharged survivor patients were categorized into two groups: the low FAR group (FAR ≤ 0.09) and the high FAR group (FAR > 0.09) using the FAR discriminative cut-off value of 0.09. *The model was adjusted for age, Glasgow Coma Scale score, hematoma location, hematoma volume, intraventricular hematoma, chronic liver disease, and craniotomy. FAR, Fibrinogen to Albumin Ratio.

The Kaplan–Meier survival curves in Supplementary Figure S1 indicate that patients with high FAR had a notably lower long-term survival rate than those with low FAR.

**Figure 2:**
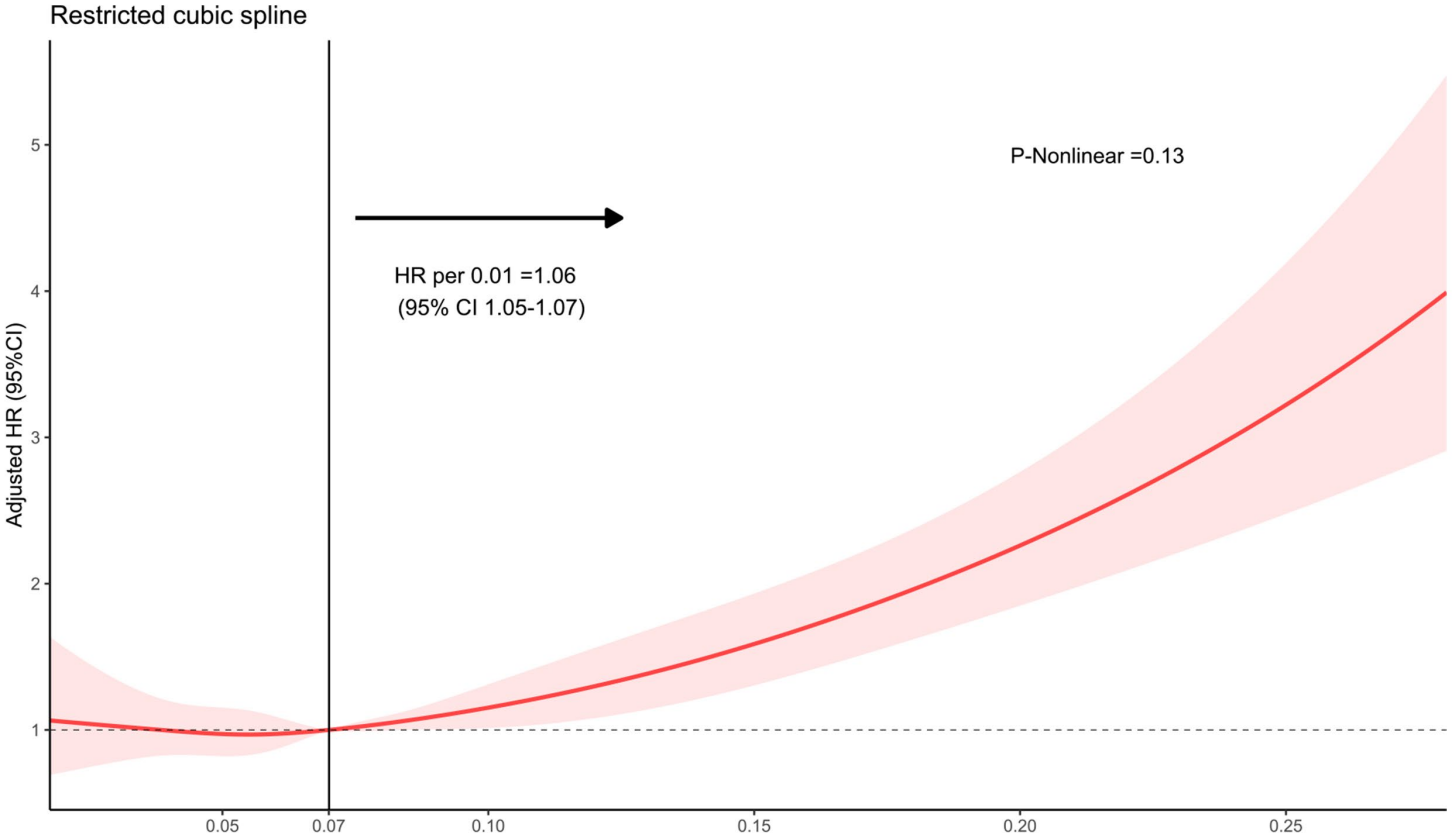
The restricted cubic spline depicting the hazard ratio of fibrinogen to albumin ratio associated with long-term mortality among patients with intracerebral hemorrhage. The x-axis represents the fibrinogen-to-albumin ratio, while the y-axis depicts the hazard ratio of long-term mortality. The model was adjusted for age, Glasgow Coma Scale score, hematoma location, hematoma volume, and intraventricular hematoma, with the FAR second quartile serving as the reference. Red indicates 95% CIs. HR, hazard ratio.

**Figure 3:**
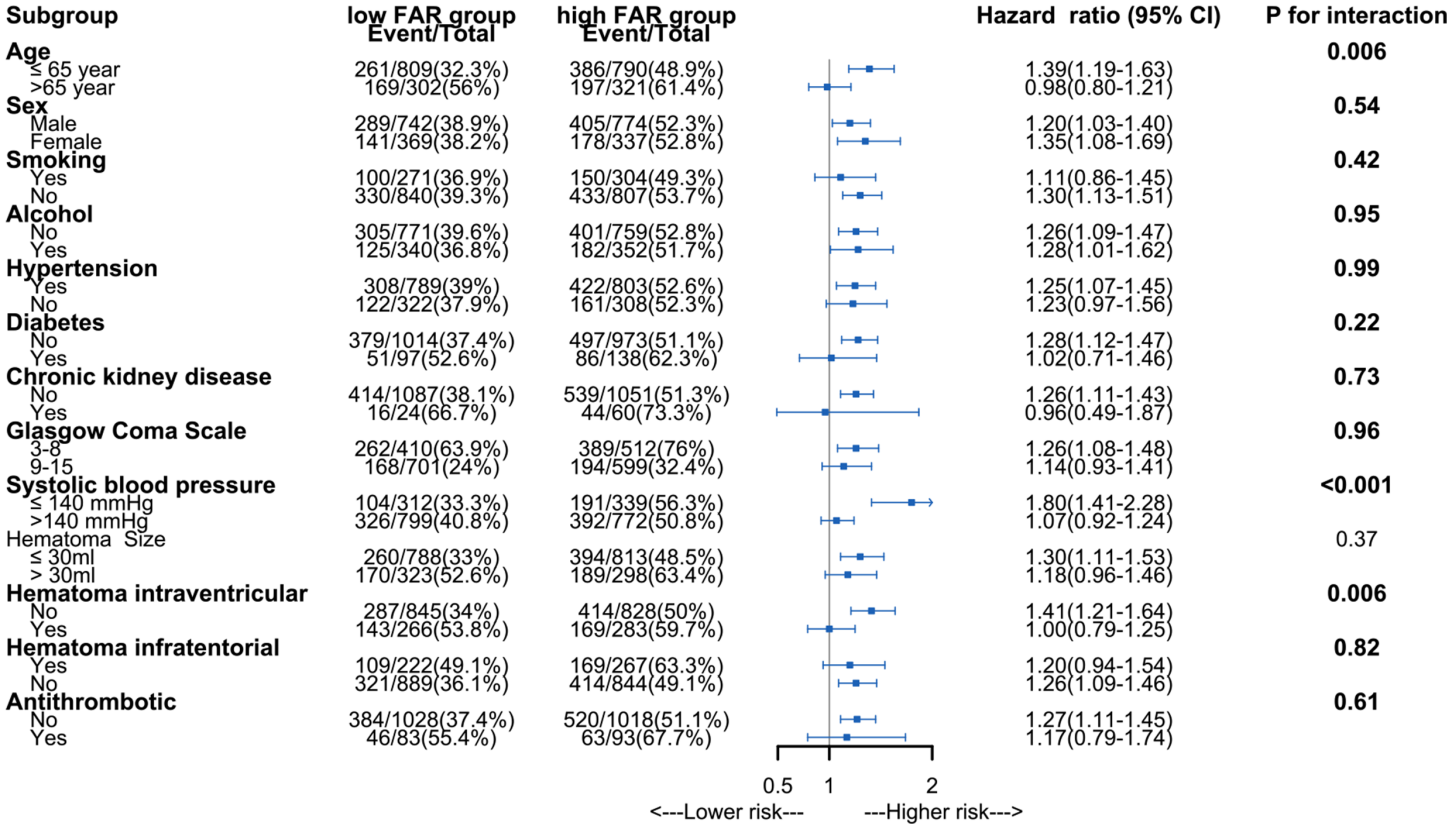
Subgroup analysis of association between fibrinogen to albumin ratio and long-term mortality.

The analysis using Restricted Cubic Splines depicted a continuous association between admission FAR and long-term mortality, as illustrated in Figure 2. Our findings indicate that higher FAR values were consistently associated with increased long-term mortality, whether patients with 1-year mortality were excluded or not. All results of the subgroup analyses are depicted in Figure 3. A significant interaction was observed between systolic blood pressure < 140 mmHg and systolic blood pressure ≥ 140 mmHg (*p* < 0.001) in all patients. Additionally, Significant interactions were also observed among age categories (≤65 years and > 65 years) and the presence of intraventricular hematoma, with both showing *p*-values lower than 0.006.

**Figure 4:**
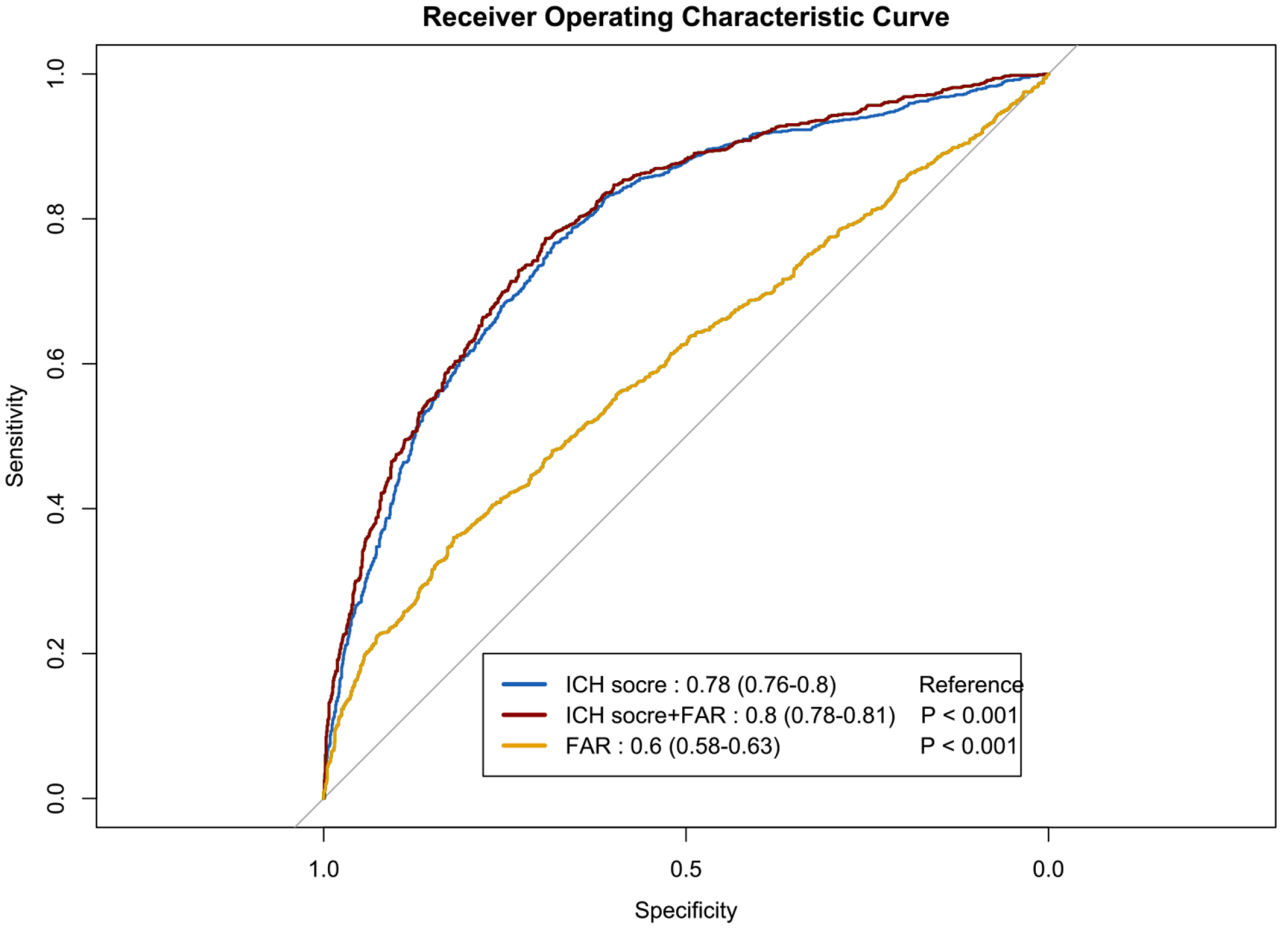
The receiver operating characteristic curves illustrating the predictive value of the FAR for long-term mortality. Model A included variables such as age, Glasgow Coma Scale score, hematoma location, hematoma volume, and intraventricular hematoma. Model B incorporated the FAR in addition to the factors in Model A. Model C solely included the FAR. ICH, Intracerebral Hemorrhage; ROC, receiver operating characteristic curve; FAR, fibrinogen to albumin ratio.

We performed ROC curve analysis to evaluate the predictive capacity of the FAR for long-term mortality and to assess a predictive model that incorporates FAR with other clinical variables (Figure 4). The ICH scores model included age, GCS score, hematoma characteristics, and intraventricular hemorrhage, resulting in an AUC of 0.78 (95% CI: 0.76–0.80; *p* < 0.001). With the addition of fibrinogen-to-albumin ratio (FAR), the AUC increased to 0.80 (95% CI: 0.78–0.81; *p* < 0.001), while FAR alone exhibited an AUC of 0.60 (95% CI: 0.58–0.63; *p* < 0.001).

## Discussion

Through our retrospective cohort study, we identified a robust association between elevated FAR at admission and long-term mortality in ICH patients. Furthermore, the combined assessment of the ICH score and FAR at admission demonstrated superior predictive accuracy for long-term mortality compared to using the ICH score alone. Our findings indicate that the FAR could potentially be a promising biomarker for risk assessment in individuals with ICH.

Two recent studies have underscored the significant role of the FAR in predicting short-term outcomes in patients with ICH. The study conducted by Bender et al. included 198 ICH patients in the intensive care unit and identified an association between FAR and in-hospital mortality (11), which aligns with our in-hospital mortality data. This indicates the potential of FAR in predicting short-term outcomes in patients with ICH. Additionally, a study by Wang et al. with 149 patients demonstrated that high FAR levels are linked to hematoma enlargement in ICH patients (10). Our study differs from previous research in several key aspects. Firstly, our research confirmed the prior findings regarding the association between FAR and in-hospital mortality. Additionally, we investigated the impact of FAR on long-term mortality outcomes in all ICH patients. Our findings revealed that FAR was linked to an elevated risk of both in-hospital and long-term mortality. Therefore, regular monitoring of FAR levels during hospitalization and follow-up appointments can offer insights into disease progression and treatment response, assisting healthcare providers in adjusting management plans to enhance long-term prognosis. Secondly, we evaluated the combined effect of the ICH score and FAR in predicting long-term mortality. The inclusion of FAR increased the AUC from 0.78 to 0.80, enhancing the predictive capacity of the model in identifying long-term mortality risk. Although the improvement from FAR was subtle, the increase was statistically significant (*p* < 0.001). Despite FAR’s limited individual predictive value (AUC of 0.60), its integration into the multidimensional ICH scores model refined risk assessment and improved overall predictive performance. Clinicians may consider utilizing FAR measurement as an additional prognostic indicator to refine risk assessment and treatment strategies.

Our findings suggest that the association of FAR and long-term mortality with ICH may be explained by various mechanisms. FAR could serve as a new inflammatory biomarker, as inflammation has been shown to significantly impact outcomes in ICH (6, 7). Previous study found that fibrinogen was associated with neuroinflammation (24, 25) and impacted long-term outcomes in patients (20, 26), Albumin, on the other hand, exhibits antioxidant and anti-inflammatory properties that protect against secondary brain injuries post-ICH (27–29) and reduce complications leading to higher mortality rates in these patients (30, 31). Prior studies by X. Wang et al. indicated that FAR >1.0 increased the risk of short- and 1-year all-cause death in acute ischemic stroke patients (32). Bender et al. also found an association between FAR and in-hospital mortality (11). Our study validated these findings and discovered an association between FAR and long-term mortality in one-year survivors of ICH. Additionally, we demonstrated that incorporating FAR improved the predictive ability of the ICH score model for identifying long-term mortality risk.

Our study highlights significant implications for clinical practice by emphasizing the potential of the FAR as a prognostic marker for long-term mortality in patients with ICH. Incorporating FAR assessment in the initial evaluation and risk stratification offers clinicians valuable insights. Evaluating FAR in conjunction with established prognostic factors such as the ICH score allows for improved assessment of illness severity and personalized interventions to optimize patient outcomes. Early identification of elevated FAR levels can assist clinicians in implementing targeted therapeutic strategies to enhance patient prognosis and reduce the risk of long-term mortality.

There are some limitations in our study. Firstly, the study’s retrospective design may introduce biases that impede establishing causality between FAR and long-term mortality in patients with ICH. Secondly, data collected from a single center may limit the generalizability of findings to a broader, more diverse population. Thirdly, our study did not consider potential changes in these biomarkers post-treatment, which could also impact the long-term mortality risk in patients with spontaneous ICH. Fourthly, confounders may have influenced the results; for instance, we cannot completely rule out the possibility of FAR elevation due to pneumonia and other infections occurring before admission. Further prospective studies are essential to confirm the results and enhance the comprehension of the link between FAR and long-term mortality in this patient cohort.

## Conclusion

A high admission FAR was significantly correlated with an elevated HR for long-term mortality in patients with ICH. The combined assessment of the ICH score and FAR at admission showed higher predictive accuracy for long-term mortality than using the ICH score in isolation. Our results suggest the clinical relevance of monitoring FAR in patients with spontaneous ICH for prognostication and potentially guiding treatment strategies to improve long-term outcomes. Further prospective studies are essential to confirm the results and investigate the underlying mechanisms of this association.

## Data availability statement

The original contributions presented in the study are included in the article/Supplementary material, further inquiries can be directed to the corresponding author.

## Ethics statement

The studies involving humans were approved by the Ethics Committee of West China Hospital (Approval number: 2022-705). The studies were conducted in accordance with the local legislation and institutional requirements. Written informed consent for participation was not required from the participants or the participants’ legal guardians/next of kin in accordance with the national legislation and institutional requirements.

## Author contributions

SC: Data curation, Formal analysis, Writing – original draft. YZ: Conceptualization, Data curation, Writing – review & editing. YX: Data curation, Formal analysis, Funding acquisition, Writing – original draft. XC: Data curation, Writing – review & editing. LP: Data curation, Formal analysis, Writing – review & editing. YT: Data curation, Investigation, Writing – review & editing. TL: Data curation, Writing – review & editing. JH: Data curation, Methodology, Writing – review & editing. PH: Data curation, Methodology, Writing – review & editing. WC: Methodology, Writing – review & editing. YH: Methodology, Supervision, Writing – review & editing. CY: Formal analysis, Writing – review & editing. FF: Formal analysis, Supervision, Writing – review & editing.
