# Supplementary material for: Association of high fibrinogen to albumin ratio with long-term mortality in patients with spontaneous intracerebral hemorrhage
Source: Front Neurol. 2024 Jul 19;15:1412804. doi: 10.3389/fneur.2024.1412804 (PMC11294216; doi:10.3389/fneur.2024.1412804)
Supplement: Supplementary file 1 [file Data_Sheet_1.docx]

**Supplemental Data**

**Table S1 Factors Associated with Long-term Mortality for all Patients.**

SBP: systolic blood pressure; GCS: Glasgow Coma Scale; NA: not available

**Table S2 Factors Associated with Long-term Mortality for Patients in 1-year Survivors.**

SBP: systolic blood pressure; GCS: Glasgow Coma Scale; NA: not available

**Table S3 The Association between Albumin and Mortality Using Multivariate Cox Regression.**

*The model was adjusted for age, Glasgow Coma Scale score, hematoma location, hematoma volume, intraventricular hematoma, chronic liver disease, and craniotomy.

Based on the ROC analysis for long-term mortality, the patients were categorized into two groups using the albumin discriminative cut-off value of 36.65 g/L

**Table S4 The Association between Fibrinogen and Mortality Using Multivariate Cox Regression.**

*The model was adjusted for age, Glasgow Coma Scale score, hematoma location, hematoma volume, intraventricular hematoma, chronic liver disease, and craniotomy.

Based on the ROC analysis for long-term mortality, the patients were categorized into two groups using the fibrinogen discriminative cut-off value of 3.25 g/L

**Figure S1 Kaplan-Meier Estimates for Long-term Survival for Patients with Intracerebral Hemorrhage.**

**Table S1 Factors Associated with Long-term Mortality for all Patients.**

| Characteristics | Univariable | | Multivariable | |
| --- | --- | --- | --- | --- |
|  | HR (95% CI) | P | HR (95% CI) | P |
| Demographics |  |  |  |  |
| Age, years | 1.02(1.02-1.02) | <0.001 | 1.02(1.01-1.02) | <0.001 |
| Female | 0.97(0.85-1.11) | 0.707 | NA | NA |
| Smoking | 0.93(0.80-1.07) | 0.29 | NA | NA |
| Alcohol | 0.95(0.83-1.09) | 0.488 | NA | NA |
| Medical history |  |  |  |  |
| Hypertension | 1.03(0.90-1.18) | 0.646 | NA | NA |
| Diabetes | 1.39(1.16-1.67) | <0.001 | NA | NA |
| Chronic kidney disease | 2.02(1.55-2.62) | <0.001 | NA | NA |
| Chronic liver disease | 1.45(1.10-1.92) | 0.009 | 1.03(0.78-1.36) | 0.604 |
| SBP | 1.00(1.00-1.00) | 0.693 | NA | NA |
| DBP | 1.00(1.00-1.00) | 0.52 | NA | NA |
| Hematoma characteristics |  |  |  |  |
| Infratentorial hematoma | 1.58(1.38-1.81) | <0.001 | 1.57(1.36-1.81) | <0.001 |
| Size of hematoma | 1.01(1.00-1.01) | <0.001 | 1.01(1.01-1.01) | <0.001 |
| Intraventricular hematoma | 1.59(1.39-1.82) | <0.001 | 1.15(1.00-1.32) | 0.053 |
| GCS score |  |  |  |  |
| 3-8 score | 1 [Reference] |  | 1 [Reference] |  |
| 9-12 score | 0.30(0.25-0.35) | <0.001 | 0.32(0.27-0.37) | <0.001 |
| 13-15 score | 0.20(0.17-0.24) | <0.001 | 0.18(0.15-0.22) | <0.001 |
| Craniotomy | 0.72(0.63-0.83) | <0.001 | 0.51(0.44-0.59) | <0.001 |
| Laboratory tests |  |  |  |  |
| Platelet | 1.00(1.00-1.00) | <0.001 | NA | NA |
| Lymphocyte count | 0.78(0.69-0.87) | <0.001 | NA | NA |
| Activated partial thromboplastin time | 1.02(1.01-1.02) | <0.001 | NA | NA |
| Blood glucose | 1.12(1.10-1.13) | <0.001 | NA | NA |
| Neutrophil count | 1.00(1.00-1.00) | <0.001 | NA | NA |
| Admission Fibrinogen to Albumin Ratio | 1.45(1.28-1.64) | <0.001 | 1.21(1.07-1.38) | 0.003 |

SBP: systolic blood pressure; DBP: Diastolic Blood Pressure; GCS: Glasgow Coma Scale.

**Table S2 Factors Associated with Long-term Mortality for Patients in 1-year Survivors.**

| Characteristics | Univariable | | Multivariable | |
| --- | --- | --- | --- | --- |
|  | HR (95% CI) | P | HR (95% CI) | P |
| Demographics |  |  |  |  |
| Age, years | 1.04(1.03-1.05) | <0.001 | 1.05(1.04-1.06) | <0.001 |
| Female | 1.05(0.81-1.36) | 0.715 | NA | NA |
| Smoking | 1.01(0.76-1.32) | 0.97 | NA | NA |
| Alcohol | 0.94(0.72-1.22) | 0.633 | NA | NA |
| Medical history |  |  |  |  |
| Hypertension | 1.24(0.94-1.63) | 0.131 | NA | NA |
| Diabetes | 2.09(1.51-2.89) | <0.001 | NA | NA |
| Chronic kidney disease | 2.06(1.12-3.76) | 0.019 | NA | NA |
| Chronic liver disease | 1.33(0.74-2.37) | 0.338 | 1.29(0.72-2.32) | 0.399 |
| SBP | 1.00(1.00-1.01) | 0.06 | NA | NA |
| DBP | 1.00(0.99-1.00) | 0.512 | NA | NA |
| Hematoma characteristics |  |  |  |  |
| Infratentorial hematoma | 1.03(0.75-1.41) | 0.844 | 1.18(0.85-1.62) | 0.322 |
| Size of hematoma | 1.01(1.00-1.01) | <0.001 | 1.00(1.00-1.01) | 0.007 |
| Intraventricular hematoma | 1.53(1.17-2.01) | 0.002 | 1.21(0.91-1.60) | 0.194 |
| GCS score |  |  |  |  |
| 3-8 score | 1 [Reference] |  | 1 [Reference] |  |
| 9-12 score | 0.40(0.30-0.55) | <0.001 | 0.41(0.30-0.56) | <0.001 |
| 13-15 score | 0.35(0.26-0.46) | <0.001 | 0.35(0.26-0.48) | <0.001 |
| Craniotomy | 1.16(0.91-1.49) | 0.235 | 1.00(0.76-1.32) | 0.998 |
| Laboratory tests |  |  |  |  |
| Platelet | 1.00(1.00-1.00) | <0.001 | NA | NA |
| Lymphocyte count | 0.78(0.69-0.87) | <0.001 | NA | NA |
| Activated partial thromboplastin time | 1.02(1.01-1.02) | <0.001 | NA | NA |
| Blood glucose | 1.12(1.10-1.13) | <0.001 | NA | NA |
| Neutrophil count | 1.00(1.00-1.00) | <0.001 | NA | NA |
| Admission Fibrinogen to Albumin Ratio | 1.47(1.15-1.88) | 0.002 | 1.31(1.02-1.68) | 0.034 |

SBP: systolic blood pressure; DBP: Diastolic Blood Pressure; GCS: Glasgow Coma Scale.

**Table S3 The Association between Albumin and Mortality Using Multivariate Cox Regression.**

| Outcomes | Events/Total, n (%) | Cox regression Unadjusted HR | P | Cox regression  Adjusted HR* | P |
| --- | --- | --- | --- | --- | --- |
| 1-year mortality |  |  |  |  |  |
| albumin ≤ 36.65g/L | 350/790(44.3%) | 1 [Reference] |  | 1 [Reference] |  |
| albumin > 36.65g/L | 400/1432(27.9%) | 0.56(0.49-0.65) | <0.001 | 0.65(0.56-0.76) | <0.001 |
| Long-term Mortality in all patients |  |  |  |  |  |
| albumin ≤ 36.65g/L | 479/790(60.6%) | 1 [Reference] |  | 1 [Reference] |  |
| albumin > 36.65g/L | 534/1432(37.3%) | 0.53(0.47-0.60) | <0.001 | 0.61(0.54-0.70) | <0.001 |
| Long-term Mortality in 1-year survivors |  |  |  |  |  |
| albumin ≤ 36.65g/L | 129/440(29.3%) | 1 [Reference] |  | 1 [Reference] |  |
| albumin > 36.65g/L | 134/1032(13%) | 0.44(0.35-0.56) | <0.001 | 0.52(0.41-0.68) | <0.001 |

*The model was adjusted for age, Glasgow Coma Scale score, hematoma location, hematoma volume, intraventricular hematoma, chronic liver disease, and craniotomy.

Based on the ROC analysis for long-term mortality, the patients were categorized into two groups using the albumin discriminative cut-off value of 36.65 g/L

**Table S4 The Association between Fibrinogen and Mortality Using Multivariate Cox Regression.**

| Outcomes | Events/Total, n (%) | Cox regression Unadjusted HR | P | Cox regression  Adjusted HR* | P |
| --- | --- | --- | --- | --- | --- |
| 1-year mortality |  |  |  |  |  |
| fibrinogen ≤ 3.25g/L | 426/1418(30%) | 1 [Reference] |  | 1 [Reference] |  |
| fibrinogen > 3.25g/L | 324/804(40.3%) | 1.45(1.25-1.67) | 0 | 1.15(0.99-1.33) | 0.069 |
| Long-term Mortality in all patients |  |  |  |  |  |
| fibrinogen ≤ 3.25g/L | 579/1418(40.8%) | 1 [Reference] |  | 1 [Reference] |  |
| fibrinogen > 3.25g/L | 434/804(54%) | 1.45(1.28-1.64) | 0 | 1.22(1.07-1.38) | 0.003 |
| Long-term Mortality in 1-year survivors |  |  |  |  |  |
| fibrinogen ≤ 3.25g/L | 153/992(15.4%) | 1 [Reference] |  | 1 [Reference] |  |
| fibrinogen > 3.25g/L | 110/480(22.9%) | 1.46(1.14-1.86) | 0.003 | 1.45(1.12-1.86) | 0.004 |

*The model was adjusted for age, Glasgow Coma Scale score, hematoma location, hematoma volume, intraventricular hematoma, chronic liver disease, and craniotomy.

Based on the ROC analysis for long-term mortality, the patients were categorized into two groups using the fibrinogen discriminative cut-off value of 3.25 g/L


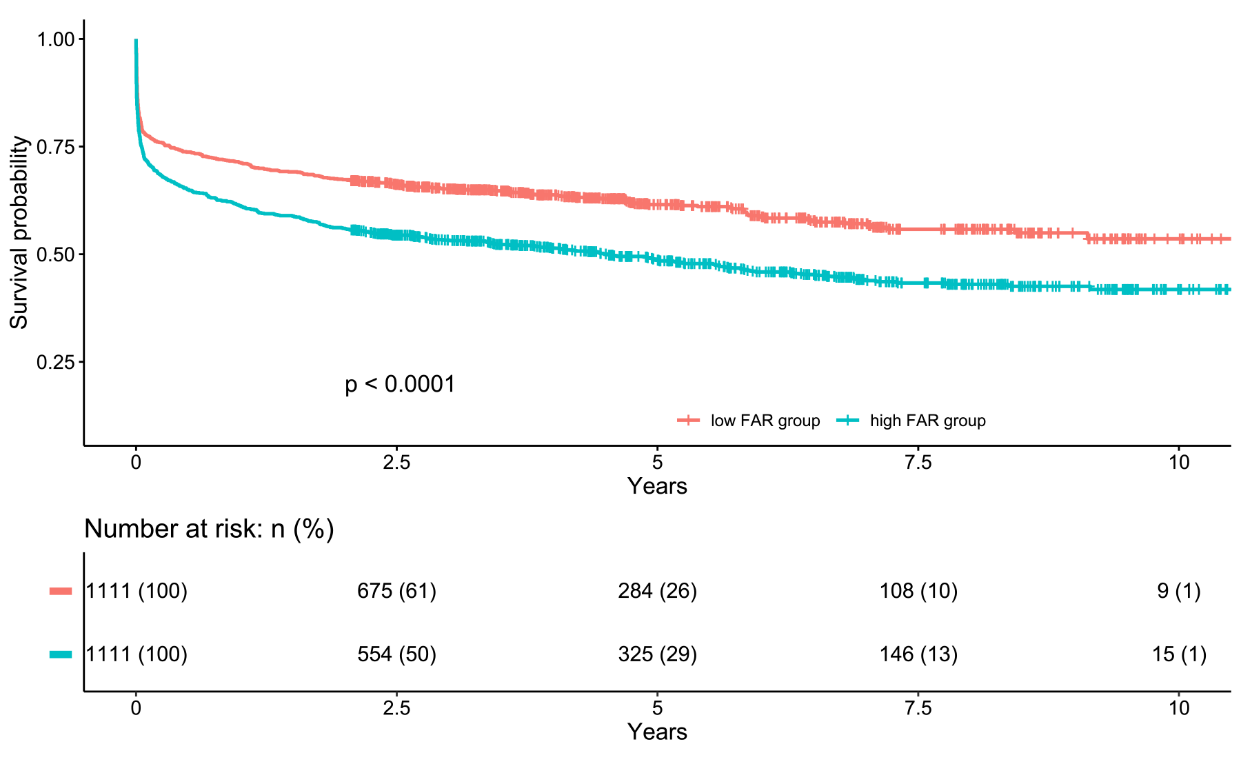


**Figure S1 Kaplan-Meier Estimates for Long-term Survival for Patients with Intracerebral Hemorrhage.**
